# Scene-Specialized Multitarget Detector with an SMC-PHD Filter and a YOLO Network

**DOI:** 10.1155/2022/1010767

**Published:** 2022-04-28

**Authors:** Qianli Liu, Yibing Li, Qianhui Dong, Fang Ye

**Affiliations:** College of Information and Communication Engineering, Harbin Engineering University, Harbin 150001, China

## Abstract

You only look once (YOLO) is one of the most efficient target detection networks. However, the performance of the YOLO network decreases significantly when the variation between the training data and the real data is large. To automatically customize the YOLO network, we suggest a novel transfer learning algorithm with the sequential Monte Carlo probability hypothesis density (SMC-PHD) filter and Gaussian mixture probability hypothesis density (GM-PHD) filter. The proposed framework can automatically customize the YOLO framework with unlabelled target sequences. The frames of the unlabelled target sequences are automatically labelled. The detection probability and clutter density of the SMC-PHD filter and GM-PHD are applied to retrain the YOLO network for occluded targets and clutter. A novel likelihood density with the confidence probability of the YOLO detector and visual context indications is implemented to choose target samples. A simple resampling strategy is proposed for SMC-PHD YOLO to address the weight degeneracy problem. Experiments with different datasets indicate that the proposed framework achieves positive outcomes relative to state-of-the-art frameworks.

## 1. Introduction

Learning-based detection algorithms have proven important in several subject areas, including smart surveillance systems [1], wireless sensors [2, 3], and secure transportation systems [4]. Over the past several years, convolutional neural networks (CNNs) have achieved excellent results in multiple computer vision assignments. You only look once (YOLO) is an effective visual detection method [5]. Compared with other detection networks, the YOLO network can predict class probabilities and bounding boxes in an assessment directly from the input frame. YOLO detectors, however, are taught with annotated datasets and utilized to attain the highest variability of the target. The distribution of the target captured by the camera may not be a subset of the initial learning set when these detectors are applied to a specific scene, such as in the case of a closed-circuit television (CCTV) camera. Therefore, the resulting Generic YOLO detector may not function effectively, especially for a limited amount of training data [6].

To address this problem, transfer learning with cross-domain adaptation is proposed. A specific training dataset is needed to generate a specific detector. Normally, these positive samples of the specific training dataset are manually selected from the target dataset. However, a large amount of labelled data is needed to tune the detector in each frame, and labelling is a labor-intensive task. A typical solution for reducing the collection time is to automatically provide the sample labels with the target frame. Labelled samples are iteratively collected from the unlabelled sequence and added to the training dataset [7].

**Figure 1:**
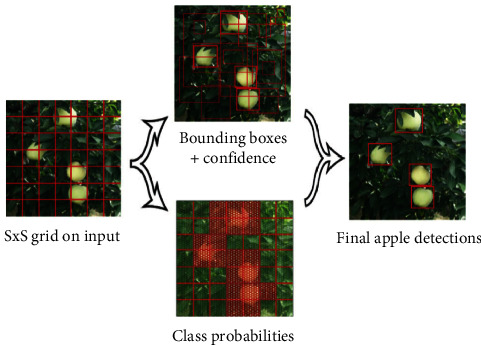
YOLO network. The red line shows the grids of images, and the red box shows the bounding boxes. The pattern-filled boxes show the grids with high probabilities.

We propose a novel transfer learning method with a probability hypothesis density (PHD) filter, which can automatically retrain a YOLO network for a special object. The scene-specific detector is generated with a Generic YOLO detector trained by labelled frames and sequences without labelled information. The parameters of the YOLO detector are estimated by an iterative process. After automatic and iterative training, the final specialized YOLO detector is produced and can run without the SMC-PHD filter.  Figure 1 illustrates the structure of our method.

Although improving the YOLO with the SMC method has been employed for transfer learning [8], the detection probability and clutter density are not considered in the target sequence. In the updated step of our proposed method, the occluded targets are selected and collected as positive samples for training. The primary benefit of our method is that the recognition model can learn the appearance of occluded targets and clutter. As shown in the experimental results in Section 4, our proposed SMC-PHD YOLO can detect some occluded speakers with the SMC-PHD filter-based occlusion strategy, while the SMC Faster region-based CNN (R-CNN) [8] cannot detect the occluded targets. In addition, when positive samples are collected, some false samples (clutter) may be added to the positive training dataset. The performance of the SMC Faster R-CNN [8] would be affected by the clutter. When there is clutter in the training dataset, the SMC Faster R-CNN produces false detection. Based on the clutter density, this clutter would be assigned a low weight, and our proposed method could disregard false samples. Our proposed PHD YOLO network has four main contributions:

- To address the bias between the training dataset and target set, we propose a PHD based transfer learning method for YOLO. For nonlinear tasks, a scene-specialized multitarget detector, SMC-PHD YOLO, is proposed. For linear systems and Gaussian noise tasks, we extend our method to GM-PHD YOLO to eliminate concerns about SMC dependence.
- In SMC-PHD YOLO, we show that the detection probability and clutter density of the SMC-PHD filter improve the performance of the retrained YOLO networks for the occluded targets and multiscale targets. When the image quality of the target scenes is unsatisfactory, even with noise, the specialized YOLO network can still detect the target with the posterior density.
- A novel likelihood is proposed to verify the selected samples in PHD YOLO. To collect positive samples for training, the confidence probability of the YOLO detector and visual context indications are applied.
- For the weight degeneracy problem of SMC YOLO, we also propose a novel and simple resampling strategy that can collect samples from the target sequence based on their weights, and the proposed distribution is assumed to be the target distribution. With the detection distribution, the strategy can function effectively even when a small number of samples is employed.

The remainder of this document is structured as follows: Section 2 introduces the current approach applied in this sector and offers details regarding the benefits of our proposed method over other specialization methods.  Section 3 describes our proposed strategy in detail. Section 4 details the configuration of the simulation and presents experimental outcomes, and concluding comments are provided in Section 5. We adhere to the convention that scale variables, such as confidence, are presented in lowercase italics, e.g., *f*. Symbols for vector-formed states and their densities are shown in lowercase bold italics, e.g., **x**, and multitarget states are represented by uppercase bold italics, e.g., **X**. Uppercase nonbold letters represent polynomials. Symbols for matrices, such as the transition matrix, are shown in uppercase bold letters, e.g., **F**.

## 2. Background

### 2.1. Specialization Frameworks

If the distribution of the training samples is different from that of target scenes, then a traditional visual detector may not function effectively [9]. To address this problem, specialization frameworks are utilized to automatically create scene-specific detectors for a target scene. Transfer learning algorithms based on state-of-the-art theories use the annotated model and expertise gained through prior assignments. There are three main types of transfer learning methods [10]. First, by changing the parameters of the source learning model, the model is improved in a target domain [11, 12]. Second, the variation between the source and target distributions is decreased, and the source learning model is adapted to the target domain [13, 14]. Third, the training samples are manually or automatically chosen, and the model is retrained with a subset of selected samples [15]. We focus on the third category because it can automatically label the selected samples and the training parameters remain unchanged.

However, the new training dataset may contain some incorrectly labelled samples because the labels of the samples are not manually verified. With this type of dataset, the accuracy of the detection framework may decrease. To address this problem, various contextual indications, such as the visual appearance of objects, pedestrian movement, road model, size, and place, are used to verify favourable samples for retraining the training dataset; however, this method is sensitive to occlusion [16]. Moreover, some techniques may only use samples from the target domain and waste helpful samples [17]. Htike and Hogg employed a background subtraction algorithm to train a particular detector [9] to select the target samples from the source and target datasets. To automatically label target information, tracklet chains are utilized to link the proposed samples to tracklets [15] predicted by an appearance-target detector. However, for each target scene, this framework, which includes many manual parameters and thresholds, may affect the specialization performance. Alternatively, Maâmatou et al. [10] collected fresh samples. To train a fresh dedicated retrained sensor, an SMC transfer learning method was employed to create a new dataset [8].

### 2.2. YOLO Network

In this work, we used the YOLO (V3) network [5] since it passes the image only once into a fully CNN (FCNN), which enables it to achieve real-time performance. YOLO (V3) was developed based on YOLO [18] and YOLO (V2) [19]. The YOLO network considers the detection problem as a regression problem. Therefore, the network directly generates a bounding box for each class via regression without any proposal region, which decreases the computational cost compared to Faster R-CNN.

The YOLO detection model is shown in Figure 1, where the network divides each input image of the training set into *S* × *S* grids. When the grid is filled by the centre of the target ground truth, the grid is used to detect the object. For each grid, several bounding boxes and their confidence scores are predicted. The confidence *f*_*s*_ is defined as

(1) $$\begin{array}{c} f_{s}=p_{r}\times {\text{IoU}}_{\text{pred}}^{\text{truth}},\;p_{r}\in \left\{0,1\right\}. \end{array}$$

If the target is in the grid, *p*_*r*_=1; otherwise, *p*_*r*_=0. IoU_pred_^truth^ (intersection over the union of the prediction and ground truth) is used to present the coincidence between the predicted bounding box and the reference bounding box, which indicates whether the grid contains targets. If several bounding boxes detect the same target, then nonmaximum suppression (NMS) is applied to select the best bounding box.

YOLO has a lower computational cost than Faster R-CNN; however, it has more errors. To address this problem, YOLO uses the “anchor” of the Faster R-CNN to generate suitable prior bounding boxes; YOLO uses k-means cluttering. The adoption of the anchor boxes decreases the mean average precision (mAP). In addition, unlike YOLO, YOLO-V3 uses batch normalization, multiscale prediction, a high-resolution classifier, dimension clutter, direct location prediction, fine-grained features, multiscale training, and other methods that greatly improve the detection accuracy.

### 2.3. Random Finite Set and PHD Filters

In this subsection, we discuss the random finite set and PHD filters for scene-specialized transform learning. The probability hypothesis density and random finite set are proposed for multitarget tracking [20–22]. The random finite set is a flexible algorithm that can be combined with any object detector to generate positional and dimensional information on objects of interest. Maggio et al. used detectors such as background subtraction, AdaBoost classifiers, and a statistical change detector to track objects associated with a random finite set (RFS) [23, 24]. For handling occlusion problems during tracking, Kim et al. proposed the labelled RFS [25]. As the RFS is a computationally expensive approximation of the multidistribution Bayes filter, the PHD is the first-order moment of the RFS, which is a set of random variables (or vectors) with random cardinality [20]. An alternative derivation of the PHD filter based on classical point process theory was given in [26]. In multitarget research, the Gaussian mixture PHD (GM-PHD) filter [27] and SMC-PHD filter [28] are widely utilized. The GM-PHD filter is a closed-form solution, as it assumes that the model is linear and Gaussian. By limiting the number of considered partitions and possible alternatives, Granstrom et al. proposed a GM-PHD filter for tracking extended targets [29]. Since different objects have different levels of clutter, an N-type GM-PHD filter was proposed for real video sequences by integrating object detector information into this filter for two scenarios [30]. However, the accuracy may decrease for nonlinear problems. To address nonlinear problems, the SMC-PHD filter was proposed based on the Monte Carlo method. With the weights of the samples (particles), the SMC-PHD filter can track a varying number of unknown targets.

The PHD filter is defined as the intensity *ψ*_*k*_, which is applied to estimate the number of speakers. The PHD filter involves a prediction step and an update step that recursively propagates the intensity function. The PHD prediction step is defined as

(2) $$\begin{array}{c} \begin{array}{c} \psi_{k|k-1}\left(x_{k}\right)=\xi_{k}\left(x_{k}\right)\\ +\int \phi_{k|k-1}\left(x_{k}|x_{k-1}\right)\psi_{k-1}\left(x_{k-1}\right)\text{d}x_{k-1} \end{array} \end{array}$$

where **x** is the target bounding box state. *ξ*_*k*_(**x**_*k*_) is the intensity of the birth RFS. *ϕ*_*k|k*−1_(**x**_*k*_*| ***x**_*k*−1_) is the analogue of the state transition probability,

(3) $$\begin{array}{c} \phi_{k|k-1}\left(x_{k}|x_{k-1}\right)=p_{S,k}\left(x_{k-1}\right)f_{k|k-1}\left(x_{k}|x_{k-1}\right)\\ +\beta_{k|k-1}\left(x_{k}|x_{k-1}\right), \end{array}$$

where *p*_*S*,*k*_(**x**_*k*−1_) is the survival probability and *f*_*k|k*−1_(**x**_*k*_*| ***x**_*k*−1_) is the transition density. *β*_*k|k*−1_(**x**_*k*_*| ***x**_*k*−1_) is the intensity function of the spawn RFS with the previous state **x**_*k*−1_. The PHD update equation is given as

(4) $$\begin{array}{c} \psi_{k}\left(x_{k}\right)=\left[1-p_{D,k}\left(x_{k}\right)\right]\psi_{k|k-1}\left(x_{k}\right)\\ +\sum_{z_{k}\in Z_{k}}\frac{p_{D,k}\left(x_{k}\right)h_{k}\left(z_{k}|x_{k}\right)\psi_{k|k-1}\left(x_{k}\right)}{\kappa_{k}\left(z_{k}\right)+\int p_{D,k}\left(x_{k}\right)h_{k}\left(z_{k}|x_{k}\right)\psi_{k|k-1}\left(x_{k}\right)}, \end{array}$$

where *h*_*k*_(**z**_*k*_*| ***x**_*k*_) is the likelihood defining the probability of **z**_*k*_ given **x**_*k*_. *p*_*D*,*k*_(**x**_*k*_) is the detection probability. The intensity of the clutter RFS **C**_*k*_ is shown as *κ*_*k*_(**z**_*k*_)=*γu*(**z**_*k*_), where *γ* is the average number of Poisson clutter points per scan and *u*(**z**_*k*_) is the probability distribution of each clutter point. The PHD recursion involves multiple integrals in equations (2) and (4), which have no closed-form solution in general. To address this issue, the SMC-PHD filter has been proposed and widely utilized [28]. In the SMC-PHD filter, at time *k* − 1, the target PHD *ψ*_*k*−1_(**x**_*k*−1_) is represented by a set of particles, {**x**_*k*−1_^*i*^, *ω*_*k*−1_^*i*^}_*i*=1_^*n*_*k*−1_^, where *n*_*k*−1_ is the number of particles at *k* − 1. To the best of our limited knowledge, this article is the first study to use the PHD filter to train a scene-specialized, multitarget detector. As the number of targets is unknown in our unlabelled dataset and the sample collection is nonlinear and non-Gaussian, the SMC-PHD filter is applied to collect the unlabelled training data and customize the YOLO network.

## 3. Proposed Framework

**Figure 2:**
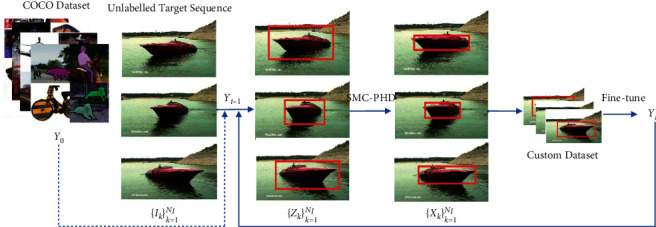
Overall framework of the proposed method. The framework input is a generic, fine-tuned YOLO detector. A visual sequence is provided to the scheme without manual labelling. To customize the YOLO network, an iterative method automatically estimates both parameters.

This section introduces our proposed framework, which customizes the YOLO model based on the PHD filter. The PHD filter is used to label the target in unlabelled videos based on the YOLO output. The positive samples estimated by the PHD filter are used to build a new custom dataset. The YOLO network is fine-tuned on this custom dataset, which may contain occluded targets and targets of different styles. Since the number of unlabelled videos is large, the bias between the training dataset and the real data decreases. Compared to the state-of-the-art method, our proposed framework is not sensitive to occlusion and target shape. The overall framework of the proposed method is shown in Figure 2.

**Figure 3:**
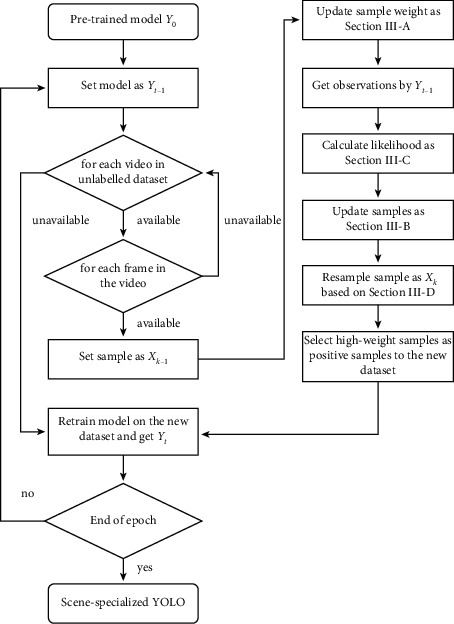
The training pipeline of the PHD YOLO detector.

To be more specific, assume that a Generic YOLO network **Y**_0_ is trained with generic datasets, such as Common Objects in Context (COCO) [31]. For the target sequence, unlabelled frames are represented as {**I**_*k*_}_*k*=1_^*n*_*I*_^, where *k* is the index of the frame. The detection output of **Y**^0^ at frame *k* is {**Z**_*k*_}_*k*=1_^*n*_*I*_^. **Z**_*k*_={**z**_*k*_^*r*^}_*r*=1_^*m*_*k*_^ is a detection set at frame *k*, where **z**_*k*_^*r*^ is a bounding box state of the detected target. *r* is the index of the detected target, and *m*_*k*_ is the number of detected targets. Furthermore, the PHD filter updates {**Z**_*k*_}_*k*=1_^*n*_*I*_^ to the estimated target state {**X**_*k*_}_*k*=1_^*n*_*I*_^. **X**_*k*_={**x**_*k*_^*j*^}_*j*=1_^*S*_*k*_^ is an estimated target set, where *b*_*k*_ is the number of estimated targets at *k* and *j* is the index of the estimated targets. Note that *n*_*I*_ is not equal to *S*_*K*_. The PHD filter removes some clutter from {**Z**_*k*_}_*k*=1_^*n*_*I*_^ and adds some missed targets. The *n*_*I*_ images with an estimated target bounding box set {**X**_*k*_}_*k*=1_^*n*_*I*_^ are applied to fine-tune the YOLO network. The fine-tuned YOLO is referred to as **Y**_*t*_, where *t* is the time of fine-tuning. The training pipeline of the PHD YOLO detector can be found in Figure 3.

The challenge is how to select the samples with the SMC-PHD filter. In this section, the iterative process is divided into three steps: prediction, updating, and resampling. In the following subsections, the details of the three primary steps are outlined. Since the SMC-PHD filter is more robust than the GM-PHD filter in the tracking task, PHD YOLO is mainly implemented as an SMC-PHD filter. To extend our proposed method to linear systems, GM-PHD YOLO is briefly discussed at the end of this section.

### 3.1. Prediction Step

To build the custom dataset, {**X**_*k*_}_*k*−1_^*n*_*I*_^, several particles are applied. At frame *k* − 1, particles are represented as **x**_*k*−1_^*i*^, *ω*_*k*−1_^*i*^, where *ω*_*k*−1_^*i*^ is the particle weight. Our work considers only two kinds of particles: survival particles and birth particles. The spawn particles of the SMC-PHD filter are disregarded. For *n*_*k*−1_ survival particles, the particle state is calculated by the transition function *F*:

(5) $$\begin{array}{c} x_{k|k-1}^{i}=Fx_{k-1}^{i}. \end{array}$$

For *b*_*k*_ birth particles, the particle state is normally set in the tracking area. The particle weight is calculated by

(6) $$\begin{array}{c} \begin{array}{c} \omega_{k|k-1}^{i}\\ =\left\{\begin{array}{cc} \frac{\phi_{k|k-1}\left(x_{k}^{i},x_{k-1}^{i}\right)\omega_{k-1}^{i}}{q_{k}\left(x_{k}^{i}|x_{k-1}^{i},Z_{k}\right)}, & i=1,\ldots ,n_{k-1},\\ \frac{\xi_{k}\left(x_{k}^{i}\right)}{b_{k}p_{k}\left(x_{k}^{i}|Z_{k}\right)}, & i=n_{k-1}+1,\ldots ,n_{k-1}+b_{k}. \end{array}\right) \end{array} \end{array}$$

However, if the new birth particle is located near the survival particles, then one target is repeatedly estimated by survival particles and birth particles. Thus, the number of targets would exceed the ground truth. To address this problem, we propose a novel birth density function based on the target state history:

(7) $$\begin{array}{c} \xi_{k}\left(x_{k}^{i}\right)=\max\left(p_{b},p_{s}\underset{\omega_{k}^{j}\in \Omega_{k}}{\max}\left(\delta_{x_{k}^{i}}\left(x_{k}^{j}\right)1_{X_{k-1}}\left(x_{k}^{j}\right)\omega_{k}^{j}\right)\right), \end{array}$$

where

(8) $$\begin{array}{c} \delta_{x_{k}^{i}}\left(x_{k}^{j}\right)=\left\{\begin{array}{cc} 1 & \text{,if }x_{k}^{i}=x_{k}^{j},\\ 0 & \text{, otherwise}, \end{array}\right)\\ 1_{X_{k-1}}\left(x_{k}^{j}\right)=\left\{\begin{array}{cc} 1 & \text{, if }x_{k}^{j}\subset X_{k-1},\\ 0 & \text{, otherwise}, \end{array}\right) \end{array}$$

where *p*_*s*_ is the survival probability and *p*_*b*_ is the birth probability. *p*_*s*_ represents the probability that the sample **x**_*k*_^*i*^ still exists. When *p*_*s*_=1, a sample still exists in the new dataset. When *p*_*s*_=0, samples are resampled, and samples in different iterations are independent.

### 3.2. Update Step

In the update step, the particle states are further updated according to the output of YOLO, {**Z**_*k*_}_*k*=1_^*n*_*I*_^. The update step of the PHD recursion is approximated by updating the weight of the predicted particles when the likelihood *h*_*k*_(**z**_*k*_*| ***x**_*k*_^*i*^) is obtained. The predicted weights are updated as

(9) $$\begin{array}{c} \begin{array}{c} \omega_{k}^{i}=\left[\left[1-p_{D}\left(x_{k}^{i}\right)\right]+\sum_{z_{k}\in Z_{k}}\frac{p_{D}\left(x_{k}^{i}\right)h_{k}\left(z_{k}|x_{k}^{i}\right)}{\kappa_{k}\left(z_{k}\right)+C_{k}\left(z_{k}\right)}\right]\omega_{k|k-1}^{i} \end{array}, \end{array}$$

where

(10) $$\begin{array}{c} C_{k}\left(z_{k}\right)=\sum_{i=1}^{n_{k-1}+b_{k}}p_{D,k}\left(x_{k}^{i}\right)h_{k}\left(z_{k}|x_{k}^{i}\right)\omega_{k|k-1}^{i}. \end{array}$$

The detection probability *p*_*D*_(**x**_*k*_^*i*^) is simplified as *p*_*D*,*k*_^*i*^ in our following work. The number of targets is estimated as the sum of the weights, *S*_*k*_=∑_*i*=1_^*n*_*k*_^*ω*_*k*_^*i*^.

To ignore the clutter, the clutter density function *κ*_*k*_(.) is applied, and the value of *κ*_*k*_(**z**_*k*_) is varied for the different detections **z**_*k*_. *κ*_*k*_(**z**_*k*_) indicates the level of clutter and is a set value. When *z*_*k*_^*r*^ has a high probability of being cluttered, *κ*_*k*_(**z**_*k*_) is a high value. If the detection is not cluttered, then *κ*_*k*_(**z**_*k*_) is given as 0. Normally, *κ*_*k*_(**z**_*k*_) is set as a constant or estimated by the Beta-Gaussian mixture model [32].

*p*_*D*_(**x**_*k*_^*i*^) is the detection probability, which is chosen based on the sample and can be estimated by the Gaussian mixture model [32]. If the sample is occluded, then *p*_*D*_(**x**_*k*_^*i*^) would have a low value (near 0). Therefore, the occluded samples have high weights and are selected for retraining the YOLO network. If the sample is not occluded, then *p*_*D*_(**x**_*k*_^*i*^) is equal to 1, and the value *h*_*k*_^*i*,*r*^ is not changed.

### 3.3. Likelihood Function

In addition to the detected probability and clutter density, the likelihood density determines whether the sample is selected for retraining. Samples with high weights are employed to retrain the YOLO network, while samples with low weights are disregarded. The likelihood density is applied to represent the relationship between the detections of the YOLO network and the samples. Therefore, we define the likelihood as

(11) $$\begin{array}{c} h_{k}=f_{s}\max\left(f_{x},\beta_{k}\right), \end{array}$$

where

(12) $$\begin{array}{c} \beta_{k}=\frac{\beta_{0}}{k}. \end{array}$$

During the iterative process, *β*_*k*_ is decreased. When the selected sample applied to retrain the YOLO detector has a high associated score, the sample likelihood is maximized. The confidence scores *f*_*s*_ are provided by the YOLO network output layer. When *f*_*s*_=0, the weight of the sample is set to 0, and the sample is removed from the specialized dataset. *f*_*x*_ indicates whether the sample was detected by the YOLO network. For visual cues, we calculate the Euclidean distance between the selected sample **x**_*k*_^*i*^ and the previous sample **X**_*k*−1_.

(13) $$\begin{array}{c} f_{x}=e^{\sum_{x_{k}^{i}\in X_{k}}D_{k}^{i,r}}\alpha_{k}^{i}, \end{array}$$

where

(14) $$\begin{array}{c} D_{k}^{i,r}={\left(u_{k}^{r}-u_{k}^{i}\right)}^{2}+{\left(v_{k}^{r}-v_{k}^{i}\right)}^{2}+{\left(w_{k}^{r}-w_{k}^{i}\right)}^{2}+{\left(h_{k}^{r}-h_{k}^{i}\right)}^{2}, \end{array}$$

where [*u*_*k*_^*r*^, *v*_*k*_^*r*^, *w*_*k*_^*r*^, *h*_*k*_^*r*^] is the state of the detection *z*_*k*_^*r*^. To select high-score samples **x**_*k*_^*i*^, we use a dynamic threshold:

(15) $$\begin{array}{c} \alpha_{k}^{i}=\left\{\begin{array}{c} \underset{x^{j}\in X_{t-1}}{\max}\delta_{x_{k}^{i}}\left(x^{j}\right)1_{X_{k-1}}\left(x^{j}\right)\delta_{y^{j}}\left(y_{k}^{i}\right)s^{j}\text{, if }k\neq 0,\\ \alpha_{0},\text{ if }k=0, \end{array}\right) \end{array}$$

where *y*^*j*^ and *y*_*k*_^*i*^ are the target class label *s* calculated by **Y**_*t*−1_. *s*_*k*_^*j*^ is the associated score, and *α*_0_ is the initial threshold.

### 3.4. Resampling Step

The SMC-PHD filter is utilized to construct a new, specific dataset for retraining, according to the resampling approach, in which resamples from the weighted dataset are included in the generated dataset {**x**_*k*_^*i*^}_*i*=1_^*n*_*k*_^. However, the traditional SMC-PHD meets the weight degeneracy problem and the number of samples decreases during the retraining step. To generate a new, unweighted dataset with the same number of samples as the weighted dataset, a sampling strategy is employed. Moreover, the effective sample size (ESS) of {**x**_*k*_^*i*^, *ω*_*k*_^*i*^}_*i*=1_^*n*_*k*_^ is calculated:

(16) $$\begin{array}{c} \text{ESS}=\frac{{\left(\sum_{i=1}^{n_{k}}\omega_{k}^{i}\right)}^{2}}{\sum_{i=1}^{n_{k}}{\left(\omega_{k}^{i}\right)}^{2}}. \end{array}$$

When the ESS is greater than 0.5, the particles can be considered to be positive samples for the special training dataset. When the ESS is less than 0.5, the particles should be resampled via the Kullback–Leibler distance (KLD) sampling [33]:

(17) $$\begin{array}{c} {\left\{x_{k}^{i}\right\}}_{i=1}^{n_{k}}\leftarrow {\left\{x_{k}^{i},\omega_{k}^{i}\right\}}_{i=1}^{n_{K}}. \end{array}$$

An extra k-means method is used to estimate **X**_*k*_ based on the particles **x**_*k*_^*i*^ _*k*=1_^*n*_*k*_^. Note that the aspect ratio of the positive training sample may differ from the initial anchors **A**_*t*−1_, as we use the IoU overlap as the positive sample. We employ the k-means method to cluster the aspect ratio of samples to update the anchors. To decrease the computational cost, only three anchors are used to retrain the YOLO network; they are set to **A**_*t*_. These proposals are employed to retrain the YOLO network, which is produced by fine-tuning the specific dataset. In the next iteration, these networks will become the input of the forecast phase and be used to create target proposals (bounding boxes) in the target scene.

### 3.5. GM-PHD YOLO

SMC-PHD is mainly discussed and applied to improve the YOLO network since it is more robust than the GM-PHD filter for nonlinear systems. However, for linear systems, the GM-PHD filter can provide a higher accuracy rate than the SMC-PHD filter. Therefore, in this subsection, we briefly discuss how to use the GM-PHD filter to improve the YOLO network. The pipeline of the GM-PHD YOLO is similar to that of SMC-PHD YOLO. YOLO is pretrained on the generic dataset, and GM-PHD assists in building the custom dataset from the unlabelled target sequences. YOLO is fine-tuned on this custom dataset. When the GM-PHD filter selects the samples, the steps include the prediction step, update step, and pruning.

In the GM-PHD filter, **x**_*k*−1_^*i*^ is distributed across the state space based on Gaussian density *N*(**m**_*k*−1_^*i*^, **P**_*k*−1_^*i*^), where **m**_*k*−1_^*i*^ and **P**_*k*−1_^*i*^ are the mean and variance, respectively. In the prediction step, for existing targets, *N* (**m**_*k*−1_^*i*^ and **P**_*k*−1_^*i*^) are predicted as **m**_*k|k*−1_^*i*^=*F ***m**_*k*−1_^*i*^ and **P**_*k|k*−1_^*i*^=**Q**+**FP**_*k*−1_^*i*^*F*^*T*^, respectively, where **Q** is the transition noise variance. Their weight is calculated as *ω*_*k|k*−1_^*i*^=*p*_*s*_*ω*_*k*_^*i*^. Birth targets are randomly chosen in the tracking area. In the update step, for undetected targets, the mean and variance retain their values, and their weights are calculated as *ω*_*k*_^*i*^=(1 − *p*_*D*_)*ω*_*k|k*−1_^*i*^. For detected targets, the mean is calculated as

(18) $$\begin{array}{c} m_{k}^{i}=m_{k|k-1}^{i}\\ +P_{k|k-1}H^{T}{\left[R+HP_{k|k-1}H^{T}\right]}^{-1}\left(z_{k}-Hm_{k|k-1}^{i}\right). \end{array}$$

The variance is updated as

(19) $$\begin{array}{c} P_{k}^{i}=\left[I-P_{k|k-1}H^{T}{\left[R+HP_{k|k-1}H^{T}\right]}^{-1}H\right]P_{k|k-1}^{i}. \end{array}$$

The particle weight is updated as

(20) $$\begin{array}{c} \omega_{k}^{i}=p_{D}\omega_{k|k-1}^{i}N\left(z_{k};Hm_{k|k-1}^{i},R+HP_{k|k-1}H^{T}\right). \end{array}$$

The weight is normalized as

(21) $$\begin{array}{c} \omega_{k}^{i}=\frac{\omega_{k}^{i}}{\kappa_{k}\left(z_{k}\right)+\sum_{i=1}^{n_{k}}\omega_{k}^{i}}. \end{array}$$

A simple pruning procedure is further employed to reduce the number of Gaussian components. The high weight targets are set to **X**_*k*_ and are utilized to build the custom dataset.

## 4. Experimental Results

This section introduces the test results obtained on several public and private datasets. First, the implementation details of our proposed method are given. Second, the dataset and baseline algorithms are introduced. Third, the ablation study of the SMC-PHD YOLO filter is discussed. Our proposed SMC-PHD YOLO detector and several baseline methods are compared.

### 4.1. Implementation Details

The initialized YOLO in our proposed SMC-PHD YOLO filter is pretrained on the COCO dataset [31]. The Adam optimizer is applied, where the weight decline is 0.0005 and the momentum is 0.9. Although the transition matrix F differs substantially across the different object classes in the different datasets, to simplify the problem, we assume F to be

(22) $$\begin{array}{c} F=\left[\begin{array}{cccc} 1 & 0 & 1 & 0\\ 0 & 1 & 0 & 1\\ 0 & 0 & 1 & 0\\ 0 & 0 & 0 & 1 \end{array}\right]. \end{array}$$

The YOLO network is fine-tuned on our evaluation dataset for the different tasks with the help of the SMC-PHD-based transforming method. The YOLO detector is tuned with a 64 GB NVIDIA GeForce GTX TITAN *X* GPU.

### 4.2. Evaluation Methodology and Dataset

We train the YOLO detector on a training collection containing 80k training frames and 500k example annotations from the COCO dataset, which contains 2.5 million labelled instances among 328k images of only 91 objects. Although the COCO dataset does not contain continuous frames, it is only used to pretrain the YOLO network before the experiments. In the evaluation step, datasets should contain continuous frames. The evaluation was performed with three different datasets.

**Figure 4:**
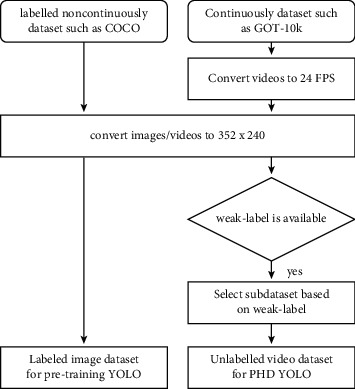
The pipeline of the data preparation for the PHD YOLO experiment.

GOT-10k [34] is a large-scale, visual dataset with broad coverage of real-world objects. It contains 10k videos of 563 categories, and its categories are more than one order of magnitude wider than those of counterparts of a similar scale. Some of its categories are not included in the COCO dataset. Therefore, GOT-10k is suitable for fine-tuning the YOLO network pretrained on the COCO dataset. The annotations that we tested include birds, cars, tapirs, and cows. YouTubeBB [35] is a large, diverse dataset with 380,000 video sections and 5.6 million human-drawn bounding boxes in 23 classifications from 240,000 distinct YouTube videos. Each video includes time-localized, frame-level features, so classifier predictions at segment-level granularity are feasible. The annotations that we tested include cars and zebras. In the MIT Traffic dataset [36], a 90-minute video is provided. A total of 420 frames from the first 45 minutes are employed for specialization, and 420 images from the last 45 minutes are utilized for testing. The video was recorded by a stationary camera. The size of the scene is 720 by 480, and it is divided into 20 clips. The annotation that we tested includes only the cars. False-positive curves per frame (FPPI) and receiver operating characteristic (ROC) curves are used to evaluate our proposed detector and baseline methods. The pipeline of the data preparation for the PHD YOLO experiment is shown in Figure 4.

### 4.3. Baseline Method

The algorithms compared with the SMC-PHD YOLO algorithm are Generic YOLO [5], Generic Faster R-CNN [37], SMC Faster R-CNN [8], that of Singh et al. [38], that of Deshmukh and Moh [39], that of Kang et al. [40], that of Maâmatou et al. [10], spatiotemporal sampling network (STSN) [41], salient object detection (SOD) [42], that of Lee et al. [43], that of Jie et al. [44], and that of Ghahremani et al. [45].  Table 1 shows the comparison between baseline methods and our method. The detector pretrained on the general dataset is presented in the second column. Some methods automatically fine-tune the network with the target dataset collected by the methods shown in the third column. For example, the algorithm of Kang et al. [40] does not include a fine-tuning step, and there is no information in its block. The computational complexity of fine-tuning with the target dataset is shown in the last column, where *n*_*I*_ is the number of frames in the video, *n* is the number of particles for the SMC method, *m* is the average number of targets in each frame, *l∗h* is the size (length ∗ width) of the frame, and *a* is the number of auxiliary networks.

### 4.4. SMC-PHD Filter YOLO for Multitarget Detection

In this subsection, we discuss the contribution of the SMC-PHD in our proposed method via three experiments. In these three experiments, we evaluate the performance of the detection probability and clutter density. Note that for a fixed label dataset and fixed YOLO, these parameters are also fixed and can be measured from the dataset. To show the contribution of the detection probability and clutter density, we set different values in the experiments.

#### 4.4.1. Detection Probability

To evaluate the detection probability performance, we set the detection probability as different constants. The detection probability in the SMC-PHD is incrementally increased from 0 to 1, and six situations are considered: 0, 0.2, 0.4, 0.6, 0.8, and 1. The YouTubeBB dataset is selected since it includes several situations. For example, the vehicles in traffic videos are frequently occluded by other vehicles, while airplanes at an airport always appear in the scene.

Table 2 shows the FPPI of the SMC-PHD YOLO network versus the detection probability and category. A correctly estimated detection probability can produce a high FPPI. For example, since the airplanes are always shown in the centre of the scene in the airplane sequences, the lowest FPPI for the airplane category is *p*_*D*,*k*_=0.2. The best results for the car category are *p*_*D*,*k*_=0.6 due to the occluded cars. Therefore, if targets are frequently occluded, then the detection probability should be of high value. Furthermore, for the airplane category, the FPPI at *p*_*D*,*k*_=1 is only 85% of that at *p*_*D*,*k*_=0.2. Thus, if the detection probability is too high, such as 1, then the FPPI of the detection would decrease.

#### 4.4.2. Clutter Density Function

The clutter density function is employed to address the clutter problem. For the PHD filter, the clutter density function is varied based on the detection results, and it is given a constant value in many references [26, 28, 32, 47]. In these experiments, clutter density is a constant value for all detections. However, a large *κ*_*k*_(**z**_*k*_^*r*^) may decrease the weights of the targets, which causes an insufficient number of samples to be included in the training dataset. A low *κ*_*k*_(**z**_*k*_^*r*^) cannot address the clutter problem, and the retrained YOLO model is still sensitive to clutter. Since *κ*_*k*_(**z**_*k*_^*r*^) is normally set to a value from 0 to infinity, we test 8 different values on the boat and bicycle sequences of the YouTubeBB dataset. Distant buildings may be detected as boats, and the bicycle detection performance is also easily affected by the surroundings. The results are shown in Table 3. The highest FPPIs for the boat sequence and bicycle sequence are 0.3 and 0.1, respectively, since the level of clutter varies for different categories. For “boat,” if *κ*_*k*_ is lower than 0.3, the FPPI would slightly decrease since clutter is added to the specialized training data and the retrained model is still sensitive to the clutter. If *κ*_*k*_ exceeds 0.3, the FPPI also decreases since the weight of the target samples decreases and the retraining dataset does not include sufficient training samples.

### 4.5. Error Analysis of the SMC-PHD YOLO Network

**Figure 5:**
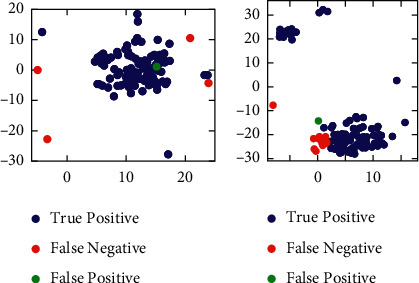
Data distribution of true positives, false positives, and false negatives for “car” and “zebra” of the YouTube BB dataset.

Since the target dataset is automatically generated by an SMC-PHD filter, it may include some error samples with uncorrected labels. To analyse whether the error samples affect the final performance, we test our SMC-PHD YOLO network with the YouTube dataset. The annotations that we employ comprise cars and zebras. The video length for each annotation, which contains 36000 frames, is 20 min. These frames are manually labelled by researchers and automatically labelled by our methods. After manually labelling these videos, 831,615 and 88,234 positive target samples were obtained for cars and zebras since multiple targets may appear in the same frame. For labels labelled by our methods, “cars” includes 797,660 true-positive samples and 212 false-positive samples, while “zebras” includes 69,821 true-positive samples and 17 false-positive samples. These results show that algorithms assign fewer labels than humans because some tiny targets and low-possibility targets are considered clutter to be disregarded. “Car” has a higher recall rate (96%) than “zebra” (79%) since cars with a regular profile are easier to detect. To further analyse these error samples, we print these data distributions. The selected features comprise the input of the last fully connected layer of YOLO. Two main dimensions are selected by t-distributed stochastic neighbour embedding.  Figure 5 shows the data distribution of true positives, false positives, and false negatives. This finding proves that tiny targets are considered to be outliers and are disregarded. We also discovered that some clutter (green points) in the target dataset is considered positive samples (false positives). After the clutter is manually disregarded in the target dataset, the YOLO performance does not change. The main potential reason for this is the high threat score (99%), and the SMC-PHD filter disregards the most uncertain samples. However, this approach does not fundamentally solve the problem of clutter since some low-possibility positive samples are considered to be false negatives (red points). Some researchers suggest the use of extra information, such as audio information, to address the clutter problem [48]. Addressing the clutter problem will be one of our future research topics.

### 4.6. Scene-Specialized Multitarget Detector

To show the performance of the PHD method for transfer learning, we compare the baseline YOLO network, SMC YOLO network, SMC R-CNN, and our proposed SMC-PHD YOLO network and GM-PHD YOLO on the YouTubeBB dataset. Since SMC R-CNN cannot address occluded samples, we propose SMC-PHD R-CNN with SMC-PHD to improve the performance of Faster R-CNN and show the effect of the PHD method. We train the YOLO network with a general training set (COCO dataset), which contains a limited amount of target data. SMC-PHD then augments a dataset containing unseen data. The unseen data in augmented data are assigned labels that may contain errors. YOLO is fine-tuned on this target dataset, and YOLO is applied without an SMC-PHD filter. The SMC-PHD filter is only applied to augment data in this work. The parameters of the PHD filter are chosen according to the Beta-Gaussian mixture model [32]. We test these methods for the airplane, bicycle, boat, and car categories of the YouTubeBB dataset. For different categories, we train the different SMC-PHD YOLO networks where parameters are independent. The YOLO network and R-CNN fine-tuned by the SMC-PHD, GM-PHD, and SMC filters are shown in Table 4. After fine-tuning YOLO, filters are not employed for target detection. Our proposed method has the highest FPPI value of all methods for the boat and car categories, and SMC-PHD YOLO performs similarly to SMC-PHD R-CNN. According to the results, SMC improves the performance of YOLO and R-CNN by approximately 8%, and PHD further improves their performance by approximately 6%. Although GM-PHD YOLO has an 8% higher FPPI than YOLO, it is still lower than that of SMC-PHD YOLO. We speculate that the reason for this is that the number of bounding boxes identified by GM-PHD YOLO is 4% more than that identified by SMC-PHD YOLO. It is proven that SMC-PHD YOLO is more robust than GM-PHD YOLO. Therefore, in the following experiment, we mainly test SMC-PHD YOLO.

**Figure 6:**
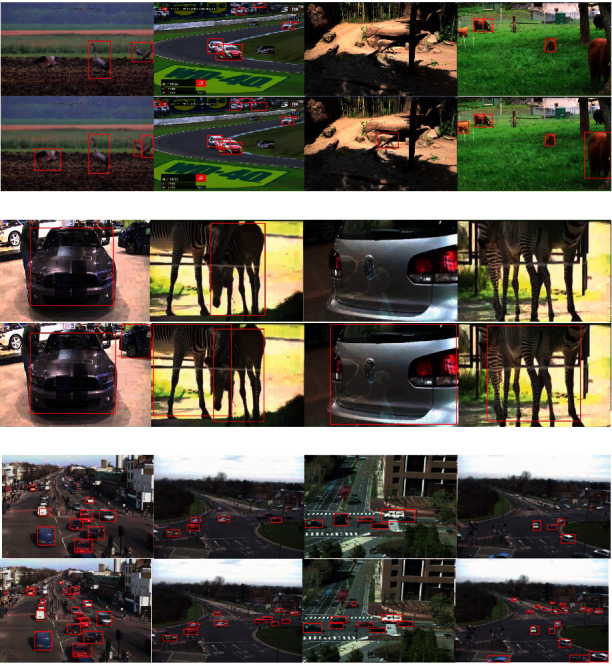
Improvement of the scene-specific detector for GOT-10k (a), YouTubeBB (b), and MIT Traffic (c). The first line of each subfigure indicates the Generic YOLO, and the second line of each subfigure indicates the SMC-PHD YOLO detector.

Some results of the proposed method and baseline methods are shown in Figure 6. The first line and second line of each subfigure are detected by Generic YOLO and specific YOLO, respectively. In Figure 6(a), the flapping bird is detected only by the specialized YOLO detectors. Thus, our proposed method can customize the detector for a moving target because the dataset is selected from a sequence with the likelihood function. In addition, some occluded cars are detected by our proposed method due to the detection probability. In Figure 6(b), cars and zebras are successfully detected by the specialized YOLO detector, even though only parts of the vehicles and zebras are shown in the images. For the traffic sequences shown in Figure 6(c), the number of cars detected with the specialized YOLO detector is higher than that detected with the Generic YOLO detector. With the SMC-PHD filter, our proposed method can detect occluded cars and certain small vehicles.

To further evaluate our proposed method, we further compare our methods with other baseline methods, such as that of Singh et al. [38], that of Deshmukh and Moh [39], that of Kang et al. [40], that of Maâmatou et al. [10], STSN [41], SOD [42], that of Lee et al. [43], that of Jie et al. [44], and that of Ghahremani et al. [45].

**Figure 7:**
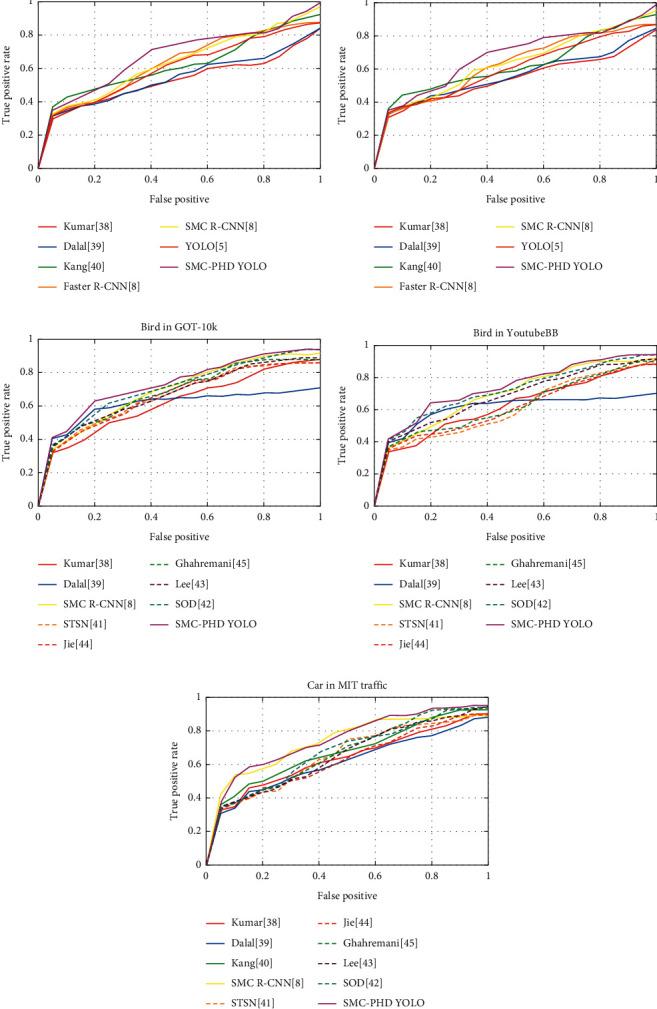
ROC curves for the Kumar, Dalal, Faster R-CNN, SMC Faster R-CNN, YOLO, and SMC-PHD YOLO methods with the bird (a) and boat (b) annotations of GOT-10k, the bird (c) and boat (d) annotations of YouTubeBB, and the car (e) annotation of the MIT Traffic dataset.

Figure 7 shows the ROC curves of the filters for the different annotations. In this experiment, we chose the bird and boat categories from the GOT-10k and YouTubeBB datasets and the car category from the MIT Traffic dataset. Due to the page limitation, Figures 7(a) and 7(b) only show a comparison between SMC-based detectors, such as SMC-PHD YOLO, and generic detectors, such as YOLO. The comparison between our proposed method and state-of-the-art methods is shown in Figures 7(c)–7(e). In Figure 7(a), the method of Kang achieves a higher true-positive rate than that of Kumar and Dalal because the former is specially designed for boat detection. Compared with the Generic YOLO for boat detection, the SMC-PHD YOLO detector achieves an ROC improvement of 13%. As the boat is often occluded in the bay, the SMC-PHD YOLO detector with the detection probability performs better than the other methods. The boat detection results on the YouTubeBB dataset are similar to those on the GOT-10k dataset. Compared with generic methods, specialized methods achieve ROC improvements of approximately 10%. More baseline transform learning methods are considered in Figure 7(c), which are shown as dashed lines. The transform methods achieve better performance than the generic R-CNN or YOLO methods. SMC based on R-CNN achieves a similar ROC value as other transform detectors. Based on SMC, the SMC R-CNN detector and SMC-PHD YOLO detector achieve increases in the ROC values of 3.8% and 5.8%, respectively, compared with their baseline methods. For car detection, we test the methods only on the MIT Traffic dataset. As shown by the ROC curves in Figure 7(e), the YOLO SMC-PHD sensor outperforms all other car detection frameworks. The SMC-PHD YOLO detector also outperforms the four other specialized detectors, i.e., SMC Faster R-CNN, that of Kumar, that of Dalal, and that of Maamatou, by 5%, 6%, 9%, and 2%, respectively.

Table 5 reports the average detection rate of our proposed method and other state-of-the-art methods for the different datasets. We list the ten annotations on GOT-10k and YouTubeBB. As the Kang and Maamatou methods are designed for boat and traffic detection, they are not included in this table. Our proposed method achieves the highest detection rate, especially for the MIT Traffic dataset. SMC-PHD YOLO can detect occluded targets, such as cars. Although SMC R-CNN achieves a detection rate similar to that of the SMC-PHD YOLO detector, the number of frames per second (FPS) of the SMC-PHD YOLO network is 100 times that of SMC R-CNN. Therefore, the SMC-PHD YOLO detector considerably outperforms the generic detector with several annotations on all government datasets. Compared to the baseline YOLO detector, the SMC-PHD YOLO detector achieves a 12% higher detection rate.

Although our proposed method has the highest detection rate and large ROC values among all methods, the proposed SMC-PHD YOLO performance depends on the hyperparameters, such as the detection probability and clutter density. These parameters should be established at the beginning of training based on previous experience. Some researchers have proposed solutions for estimating the parameters of the SMC-PHD filter. For example, Lian et al. [49] used the expectation maximum to estimate the unknown clutter probability, and Li et al. [50] used the gamma Gaussian mixture model to estimate the detection probability. Applying this kind of estimation method to improve the SMC-PHD YOLO filter will be addressed in our future work.

## 5. Conclusion

To customize the YOLO detector for unique target identification, we suggested an effective and precise structure based on the SMC-PHD filter and GM-PHD filter. On the basis of the proposed confidence score-based likelihood and novel resampling strategy, the framework can be employed by choosing appropriate samples from target datasets to train and then detect a target. This framework automatically offers a strong specialized detector with a Generic YOLO detector and some target videos. The tests showed that the proposed framework can generate a specific YOLO detector that considerably outperforms the Generic YOLO detector on a distinct dataset for bird, boat, and vehicle detection. Correlated clutter is still challenging for SMC-PHD filters. Our future research will focus on expanding the algorithm with multimodal information to address the correlated clutter problem.

## Figures and Tables

**Table 1:** Comparison between baseline methods and our method.

Baseline	Detector	Fine-tuned	Computational complexity
YOLO [5]	YOLO	—	—
R-CNN [37]	R-CNN	—	—
SMC R-CNN [8]	R-CNN	SMC	*𝒪*(*n*_*I*_*∗n∗m*)
Singh et al. [38]	R-CNN	Track and segment [46]	*𝒪*(*n*_*I*_*∗l∗h*)
Deshmukh and Moh [39]	CNN	Edge detectors	*𝒪*(*n*_*I*_*∗l∗h*)
Kang et al. [40]	Contextual R-CNN	—	—
Maâmatou et al. [10]	SVM	SMC	*𝒪*(*n*_*I*_*∗n∗m*)
STSN [41]	STSN	—	—
SOD [42]	SOD	R101 FPN	*𝒪*(*n*_*I*_*∗n∗m*)
Lee et al. [43]	R-CNN	Auxiliary network	*𝒪*(*n*_*I*_*∗n∗m∗a*)
Jie et al. [44]	R-CNN	Online supportive sample harvesting [44]	*𝒪*(*n*_*I*_*∗n∗m*)
Ghahremani et al. [45]	CNN	F1 score threshold	*𝒪*(*n*)
SMC-PHD YOLO	YOLO	SMC-PHD	*𝒪*(*n*_*I*_*∗n∗m*)

**Table 2:** FPPI of the SMC-PHD YOLO network versus detection probability for the “airplane” and “car” categories of the YouTubeBB dataset.

*p*_*D*,*k*_	0	0.2	0.4	0.6	0.8	1
Airplane	0.80	0.81	0.78	0.75	0.72	0.69
Car	0.80	0.83	0.86	0.88	0.85	0.81

**Table 3:** FPPI of the SMC-PHD YOLO network versus detection probability for the “boat” and “bicycle” categories of the YouTubeBB dataset.

*κ*_*k*_	0	0.1	0.3	0.7	0.9	1	5	10
Boat	0.81	0.82	0.84	0.82	0.74	0.68	0.58	0.51
Bicycle	0.67	0.69	0.65	0.59	0.51	0.48	0.39	0.34

**Table 4:** FPPI of our proposed SMC-PHD YOLO, SMC YOLO, YOLO, SMC-PHD R-CNN, and SMC R-CNN on the YouTubeBB dataset.

Method	Airplane	Bicycle	Boat	Car
SMC-PHD YOLO	0.81	0.69	0.84	0.88
GM-PHD YOLO	0.79	0.65	0.82	0.84
SMC YOLO	0.76	0.63	0.76	0.81
YOLO	0.71	0.57	0.68	0.76
SMC-PHD R-CNN	0.82	0.70	0.83	0.88
SMC R-CNN	0.79	0.67	0.83	0.89

**Table 5:** Detection rate for the different datasets with different detections (at 1 FPPI).

Data		SMC-PHD YOLO	YOLO [5]	SMC R-CNN [8]	Kumar [38]	Dalal [39]	STSN [41]	Jie [44]	Ghahremani [45]	Lee [43]	SOD [42]
YoutubeBB	Airplane	0.91	0.81	0.87	0.8	0.8	0.83	0.85	0.86	0.83	0.89
	Bicycle	0.89	0.77	0.86	0.78	0.75	0.82	0.83	0.82	0.84	0.87
	Bird	0.94	0.85	0.92	0.82	0.84	0.87	0.86	0.86	0.88	0.89
	Boat	0.98	0.87	0.96	0.83	0.84	0.89	0.91	0.92	0.93	0.95
	Bus	0.96	0.83	0.95	0.82	0.81	0.89	0.86	0.9	0.92	0.93
	Car	0.98	0.86	0.95	0.84	0.83	0.91	0.92	0.92	0.93	0.94
	Cat	0.94	0.85	0.92	0.82	0.83	0.93	0.91	0.93	0.92	0.95
	Cow	0.98	0.87	0.95	0.86	0.88	0.95	0.96	0.94	0.95	0.96
	Dog	0.92	0.81	0.89	0.80	0.82	0.88	0.91	0.89	0.9	0.88
	Horse	0.96	0.85	0.94	0.86	0.86	0.92	0.9	0.89	0.93	0.95

GOT-10k	Anteater	0.53	0.39	0.41	0.37	0.42	0.52	0.48	0.51	0.49	0.52
	Bird	0.94	0.88	0.92	0.87	0.79	0.86	0.86	0.88	0.89	0.93
	Cat	0.91	0.83	0.90	0.84	0.79	0.84	0.86	0.88	0.9	0.87
	Elephant	0.88	0.73	0.86	0.75	0.70	0.82	0.84	0.87	0.89	0.85
	Boat	0.98	0.87	0.97	0.84	0.84	0.87	0.89	0.92	0.94	0.97
	Goat	0.88	0.72	0.87	0.76	0.69	0.78	0.8	0.83	0.85	0.87
	Horse	0.87	0.71	0.85	0.73	0.75	0.81	0.83	0.84	0.86	0.85
	Lion	0.86	0.73	0.84	0.71	0.77	0.81	0.83	0.84	0.85	0.83
	Car	0.95	0.85	0.91	0.86	0.87	0.85	0.87	0.93	0.94	0.94
	Tank	0.74	0.61	0.68	0.63	0.61	0.63	0.66	0.69	0.71	0.73

MIT	Pedestrian	0.97	0.85	0.93	0.86	0.82	0.91	0.93	0.95	0.94	0.96
	Car	0.95	0.88	0.89	0.93	0.89	0.9	0.92	0.93	0.95	0.96

Average		0.9	0.79	0.87	0.79	0.78	0.84	0.85	0.86	0.87	0.89

## Data Availability

The data used to support this study are available from the corresponding author upon request.

## References

[1] Hampapur A., Brown L., Connell J. (Mar. 2005). Smart video surveillance: exploring the concept of multiscale spatiotemporal tracking. *IEEE Signal Processing Magazine*.

[2] Javadi S., Moosaei H., Ciuonzo D. (2019). Learning wireless sensor networks for source localization. *Sensors*.

[3] Zhao L., Huang Z. A moving object detection method using deep learning-based wireless sensor networks. *Complexity*.

[4] Thiolliere R., Dunbar E., Synnaeve G., Versteegh M., Dupoux E. (2015). A hybrid dynamic time warping-deep neural network architecture for unsupervised acoustic modeling. *Proc. INTERSPEECH*.

[5] Redmon J., Farhadi A. (2018). Yolov3: an incremental improvement. https://arxiv.org/abs/1804.02767.

[6] Dollár P., Tu Z., Perona P., Belongie S. Integral channel features.

[7] Benfold B., Reid I. Stable multi-target tracking in real-time surveillance video.

[8] Mhalla A., Chateau T., Maâmatou H., Gazzah S., Ben Amara N. E., Smc faster R.-C. N. N. (2017). SMC faster R-CNN: toward a scene-specialized multi-object detector. *Computer Vision and Image Understanding*.

[9] Htike K. K., Hogg D. C. Efficient non-iterative domain adaptation of pedestrian detectors to video scenes.

[10] Maâmatou H., Chateau T., Gazzah S., Goyat Y., Amara N. E. B. Transductive transfer learning to specialize a generic classifier towards a specific scene.

[11] Aytar Y., Zisserman A. Tabula rasa: model transfer for object category detection.

[12] Tommasi T., Orabona F., Caputo B. (2014). Learning categories from few examples with multi model knowledge transfer. *IEEE Transactions on Pattern Analysis and Machine Intelligence*.

[13] Pan S. J., Tsang I. W., Kwok J. T., Yang Q. (2011). Domain adaptation via transfer component analysis. *IEEE Transactions on Neural Networks*.

[14] Quanz B., Huan J., Mishra M. (2012). Knowledge transfer with low-quality data: a feature extraction issue. *IEEE Transactions on Knowledge and Data Engineering*.

[15] Mao Y., Yin Z. Training a scene-specific pedestrian detector using tracklets.

[16] Rosenberg C., Hebert M., Schneiderman H. Semi-supervised self-training of object detection models.

[17] All K., Hasler D., Fleuret F. Flowboost appearance learning from sparsely annotated video.

[18] Redmon J., Divvala S., Girshick R., Farhadi A. You only look once: unified, real-time object detection.

[19] Redmon J., Farhadi A. Yolo9000: better, faster, stronger.

[20] Mahler R. P. S. (2003). Multitarget bayes filtering via first-order multitarget moments. *IEEE Transactions on Aerospace and Electronic Systems*.

[21] Mahler R. (2007). Phd filters of higher order in target number. *IEEE Transactions on Aerospace and Electronic Systems*.

[22] Mahler R. P. (2007). Statistical multisource-multitarget information fusion. *Artech House Norwood*.

[23] Maggio E., Taj M., Cavallaro A. (2008). Efficient multitarget visual tracking using random finite sets. *IEEE Transactions on Circuits and Systems for Video Technology*.

[24] Maggio E., Cavallaro A. (2009). Learning scene context for multiple object tracking. *IEEE Transactions on Image Processing*.

[25] Kim D. Y., Vo B.-N., Vo B.-T. (2016). Online visual multi-object tracking via labeled random finite set filtering. https://arxiv.org/abs/1611.06011.

[26] Singh S. S., Vo B.-N., Baddeley A., Zuyev S. (2009). Filters for spatial point processes. *SIAM Journal on Control and Optimization*.

[27] Vo B.-N., Ma W.-K. (2006). The Gaussian mixture probability hypothesis density filter. *IEEE Transactions on Signal Processing*.

[28] Ba-Ngu Vo B.-N., Singh S., Boucet A. (2005). Sequential Monte Carlo methods for multi-target filtering with random finite sets. *IEEE Transactions on Aerospace and Electronic Systems*.

[29] Granstrom K., Lundquist C., Orguner O. (2012). Extended target tracking using a Gaussian-mixture phd filter. *IEEE Transactions on Aerospace and Electronic Systems*.

[30] Baisa N. L., Wallace A. (2019). Development of a N-type GM-PHD filter for multiple target, multiple type visual tracking. *Journal of Visual Communication and Image Representation*.

[31] Lin T.-Y., Maire M., Belongie S. Microsoft coco: Common objects in context.

[32] Mahler R. P. S., Vo B.-T., Vo B.-N. (2011). Cphd filtering with unknown clutter rate and detection profile. *IEEE Transactions on Signal Processing*.

[33] Fox D. (2002). Kld-sampling: adaptive particle filters. *Advances in Neural Information Processing Systems*.

[34] Huang L., Zhao X., Huang K. (2018). Got-10k: a large high-diversity benchmark for generic object tracking in the wild. https://arxiv.org/abs/1810.11981.

[35] Real E., Shlens J., Mazzocchi S., Pan X., Vanhoucke V. Youtube-boundingboxes: a large high-precision human-annotated data set for object detection in video.

[36] Wang X. X., Xiaoxu Ma X., Grimson W. E. L. (2009). Unsupervised activity perception in crowded and complicated scenes using hierarchical bayesian models. *IEEE Transactions on Pattern Analysis and Machine Intelligence*.

[37] Ren S., He K., Girshick R., Sun J., Faster R.-C. N. N. (2015). Towards real-time object detection with region proposal networks. *Advances in Neural Information Processing Systems*.

[38] Singh K. K., Xiao F., Jae Lee Y. Track and transfer: watching videos to simulate strong human supervision for weakly-supervised object detection.

[39] Deshmukh S., Moh T.-S. Fine object detection in automated solar panel layout generation.

[40] Kang M., Ji K., Leng X., Lin Z. (2017). Contextual region-based convolutional neural network with multilayer fusion for sar ship detection. *Remote Sensing*.

[41] Bertasius G., Torresani L., Shi J. Object detection in video with spatiotemporal sampling networks.

[42] Li Y., Huang D., Qin D., Wang L., Gong B. Improving object detection with selective self-supervised self-training. *Computer Vision - ECCV 2020*.

[43] Lee W., Na J., Kim G. Multi-task self-supervised object detection via recycling of bounding box annotations.

[44] Jie Z., Wei Y., Jin X., Feng J., Liu W. Deep self-taught learning for weakly supervised object localization.

[45] Ghahremani A., Bondarev E., de With P. H. N. (2019). Towards multi-class detection: a self-learning approach to reduce inter-class noise from training dataset. *International Society for Optics and Photonics*.

[46] Xiao F., Jae Lee Y. Track and segment: an iterative unsupervised approach for video object proposals.

[47] Li T., Sun S., Bolić M., Corchado J. M. (2016). Algorithm design for parallel implementation of the SMC-PHD filter. *Signal Processing*.

[48] Liu Y., Hilton A., Chambers J., Zhao Y., Wang W. Non-zero diffusion particle flow smc-phd filter for audio-visual multi-speaker tracking.

[49] Lian F., Han C., Liu W. (2010). Estimating unknown clutter intensity for phd filter. *IEEE Transactions on Aerospace and Electronic Systems*.

[50] Li C., Wang W., Kirubarajan T., Sun J., Lei P. (2018). Phd and cphd filtering with unknown detection probability. *IEEE Transactions on Signal Processing*.

